# Nanomodification of Lightweight Fiber Reinforced Concrete with Micro Silica and Its Influence on the Constructive Quality Coefficient

**DOI:** 10.3390/ma14237347

**Published:** 2021-11-30

**Authors:** Evgenii M. Shcherban’, Sergey A. Stel’makh, Alexey Beskopylny, Levon R. Mailyan, Besarion Meskhi, Valery Varavka

**Affiliations:** 1 Department of Engineering Geology, Bases, and Foundations, Don State Technical University, 344003 Rostov-on-Don, Russia; au-geen@mail.ru (E.M.S.); sergej.stelmax@mail.ru (S.A.S.); 2 Department of Transport Systems, Faculty of Roads and Transport Systems, Don State Technical University, 344003 Rostov-on-Don, Russia; 3 Department of Roads, Don State Technical University, 344003 Rostov-on-Don, Russia; lrm@aaanet.ru; 4 Department of Life Safety and Environmental Protection, Faculty of Life Safety and Environmental Engineering, Don State Technical University, 344003 Rostov-on-Don, Russia; reception@donstu.ru; 5 Research and Education Center “Materials”, Don State Technical University, Gagarin sq., 1, 344003 Rostov-on-Don, Russia; varavkavn@gmail.com

**Keywords:** nano-modifying, lightweight concrete, micro silica, coefficients of constructive quality, optimal composition, fiber concrete

## Abstract

A hypothesis was put forward that a nano-modifying additive of micro silica, which had a beneficial effect on achieving a perfect structure of heavy concrete, can also be effectively used in lightweight fiber-reinforced concrete. The nano-modifying additives of micro silica application in manufacturing lightweight fiber reinforced concrete products and structures can significantly enchain their strength characteristics without increasing their mass and consequently improve their design characteristics. The purpose of the work was to increase the structural quality coefficients for all types of strengths of lightweight fiber-reinforced concrete due to its modification with micro silica. The effect of nano-modifying additives of micro silica on the strength characteristics of lightweight fiber reinforced concrete was studied. The optimal amount of micro silica addition was experimentally confirmed and established of 10% of the cement mass. The coefficients of constructive quality for all experimentally determined strength characteristics of lightweight fiber-reinforced concrete modified with micro silica additives were calculated. The coefficient of constructive quality for tensile strength in bending of lightweight fiber reinforced concrete with additives was two and a half times higher than that of heavy concrete without additives and up to 37% higher than that of lightweight fiber-reinforced concrete without additives.

## 1. Introduction

Among the innovative areas that improve the efficiency of cement materials are their fine grinding, activation of water and mortars, reinforcement with fillers, etc. [1,2]. Fine grinding can be carried out in high-energy mills and rotor-pulsating units. An increase in the specific surface area of powders from 200 to 800 m^2^/kg leads to a significant increase in the strength of concrete. Water was activated by electric, magnetic fields, gravity, or other methods [1,2]. 

Considerable attention of researchers is paid to the use of production waste during new construction materials obtaining. At the same time, the following tasks are considered: environmental protection, involvement of waste in the production of building materials to reduce the consumption of cement and improve the economic performance, improving the quality of building materials, including their operational resistance and durability, creation of waste-free technologies to produce building materials. Furthermore, the structural health monitoring techniques can investigate their condition to ensure the safety of the infrastructure during its service life [3,4,5].

The finely dispersed technogenic and natural raw materials application in building manufacture is due to both factors: the need large-tonnage production wastes utilization (ash and slag mixtures, fly ash, slags, dressing waste, overburden, etc.), and the requirement to improve the quality of building materials [6,7,8]. These additives can be used to prepare binders; heavy, fine-grained, light, cellular, fire and heat-resistant, decorative and radiation-protective concretes; heat-insulating, wall, ceramic, facing, refractory products; fillers of industrial openings. In addition, mineral additives have a sealing effect on the structure of building materials, especially in multicomponent binder compositions.

The following methods can be used to increase the efficiency of cement use and reduce energy consumption in the manufacture of concrete: the inclusion of additives of micro-fillers, including micro silica and reinforcing fillers such as wollastonite; the introduction of superplasticizers; mechanical activation of cement; the impact of electromagnetic waves [9,10,11].

When non-functional organosilicon compounds and superplasticizers are used as additives, their mechanical activation is expedient. In this case, polyorganosiloxanes can enter into a chemical reaction with cement minerals, resulting in heterosiloxane structures that are resistant to the action of alkalis [12].

For example, in [13], the current state of modeling the molecular dynamics (MD) of calcium silicate hydrate (CSH) and its various modifications for nanotechnological cement composites, including carbon-based nanomaterials (for example, carbon nanotubes, graphene, graphene oxide) is comprehensively considered. It is summarized that the MD method can calculate and visualize the behavior of cement hydrates and other ingredients in a cement matrix at the nanoscale. Thus, it becomes possible to study the fundamental properties of the CSH structure and its interaction with nanoparticles [13].

Reducing the amount of clinker in cement binders is very important today, and nanotechnology allows to expand these opportunities. So, in work [14] the optimization of the binder was carried out, consisting of 60% Portland cement, 20% limestone, 20% fly ash, 3% polyvinyl alcohol (PVA) fibers, and 2% superplasticizer. Its optimization was carried out using three different nano montmorillonite (nMt) dispersions: two organically modified and one inorganic in different proportions (from 0.5% to 4%). Flexural strength, measured at 7, 28, 56 and 90 days, was improved after 28 days of hardening with the addition of inorganic nMt. Thermogravimetric analyses performed on days 7, 28, 56 and 90 combined with X-ray diffraction (on day 28) showed an enhanced pozzolanic response. Further measurements of the relative density nMt of cement nanocomposites showed higher values than that of the reference composition, which can be explained by better packing of particles. Proposals were also made for further activation of cement nanocomposites reinforced with nMt fiber. Waterproof tests have suggested that inorganic nMt may be a viable alternative material where permeability is a prerequisite [14].

The need to produce environmentally friendly cement has driven research towards nanotechnology. The main product of cement hydration—calcium silicate hydrate—is nanoscale in nature. Therefore, the addition of nanoparticles to mixed compositions of Portland cement can significantly change the mechanical strength, porosity and durability. The work [15] considers the material aspects of nanocomponents: nano-silica and montmorillonite nano clay. The disadvantages that arise when they are added to cement pastes and ways to eliminate these disadvantages were considered. It is concluded that solid particles of nano-silica in mixed cement pastes should not exceed 0.5%, and solid particles of nano clay—almost 1% of the binder mass. Finally, the authors summarize that, despite the progress made, inorganic nano clay has significant potential [15].

In [16], the effect of nano-SiO_2_(nS) particles on cement binders was studied. NS particles dispersed in (a) polycarboxylate or (b) water were added to a reference low carbon binder containing 43% Portland cement (PC), 20% limestone powder (LS) and 37% fly ash (FA) by weight binder. Eight quaternary binders containing nS, PC, LS and FA and eight quaternary binders containing nS, PC, LS, FA and micro silica (μS) were then tested. nS was added in an amount of 0.1%, 0.2%, 0.5% or 1.0% by weight of the binder as a substitute for LS for four-component binders and in an amount of 0.5% or 1.0% for five-component binders. It was concluded that, in such complex compositions, hydration products seem to create a shell around FA particles, delaying their activation at an early age. At a later age, the addition of 0.5% nS improved strength, microstructure and hydration. Polycarboxylate/nS particles provided a more pronounced improvement in strength with the addition of 0.5%, possibly due to their superplasticizing effect [16].

A study [17] evaluated the effect of adding nanoparticles of silicon dioxide (NS) to two binder matrices, such as ordinary Portland cement and sulfoaluminate cement, to establish their effect on mechanical and chemical properties. NS was added at doses ranging from 0.3 to 5.0% by weight concerning the cement to carry out this assessment. The results showed that the compressive strength and chemical resistance of sulfates were improved by adding silica nanoparticles to both matrices. Finally, resistance to the chemical attack of sulfates was shown to be enhanced with the addition of silicon dioxide nanoparticles compared to pure cement, indicating an increase in compaction [8].

In [18], an assessment was made of the effect of nano-silica on the calcium silicate hydrate network and the microstructure of hardened pastes based on Portland cement with ternary, quaternary and five-component systems. The sample obtained in the presence of micro silica and nano-silica (five-component combination) demonstrated the presence of a more complex gel network of calcium silicate hydrate (bridging tetrahedrons), characteristic of a honeycomb structure, in contrast to the triple combination (control sample) [18].

In the article [19], a study of the effect of nanoparticles on the mechanical properties of concrete of different ages was carried out. Various mixtures have been studied, including nano-silica (NS), nano clay (NC), or both NS and NC together with different percentages, and mechanical properties such as compressive and flexural strengths have been investigated. This study showed that nanoparticles can be very effective in improving the mechanical properties of concrete, nano-silica is more effective than nano clay in mechanical properties, and a wet mix is more effective than a dry mix. On the other hand, exceeding a certain percentage of nanoparticles in concrete negatively affects the mechanical properties. The combined use of nanoparticles (NS + NC) showed a marked improvement in the compressive strength of concrete than when using the same percentage of nanoparticles of the same type. This improvement can be explained by the reaction of nanomaterials with crystals of calcium hydroxide Ca(OH)_2_, which are located in the interfacial zone (ITZ) between the hardened cement paste and aggregates and form a C-SH gel and the filling action of nanoparticles, which form a more compacted microstructure. A total 3% of nanoparticles, consisting of 25% NS and 75% NC, gave the highest mechanical properties: compressive and flexural strength [19].

Polypropylene fibers with carbon nanotubes obtained by melt spinning were analyzed in [20] from the point of view of experimental mechanical properties and numerical nonlocal models. Experimental results showed that, although the applied processing conditions are such that the inclusion of carbon nanotubes does not change the crystal structure and the degree of crystallinity of the matrix-base, the tensile properties of nanocomposite fibers changed significantly depending on the filler content [20].

The effect of microstal fiber, granular light fly ash aggregate and microsilica content on the fresh and hardened properties of a high-performance binder composite (HPCC) has been experimentally investigated [21]. The mechanical and physical properties of HPCC mixtures were evaluated. The effects of fiber content, silica fume addition and artificial lightweight aggregate content were studied. The experimental results showed that the mechanical properties of the HPCC and the shrinkage characteristics improved with the increase in the volume fraction of the steel fiber. The authors summarize that the negative effect of artificial lightweight aggregate can be eliminated with silica fume [21].

Analysis of literature data [9,10,11,12,13,14,15,16,17,18,19,20,21,22,23,24,25,26,27,28,29] shows that dispersed mineral additives are widely used in the manufacture of cement building materials and products. Thus, both tasks of improving the properties of building materials and the disposal of industrial waste are being solved. At the same time, the amount of added additives in some cases is not chosen reasonably enough. The dispersion of additives, the dependence of the properties of materials based on cement on this dispersion and the amount of added additives are not always analyzed. In this regard, a study on the search for a rational formulation, dosage and interaction of micro silica with other components of heavy concrete is seen as promising.

A hypothesis was put forward that a nano-modifying additive, which has a beneficial effect on achieving a perfect structure of heavy concrete, can be simultaneously used in lightweight fiber concrete. It is assumed that the use of nano-modifying additives of micro silica in the technology of manufacturing lightweight fiber-reinforced concrete products and structures will significantly increase their strength characteristics without increasing their mass and, as a consequence, improve their design characteristics.

Scientific novelty: for the first time, studies were carried out to increase the coefficients of the constructive quality of lightweight fiber-reinforced concrete due to its modification with micro silica.

**Figure 1:**
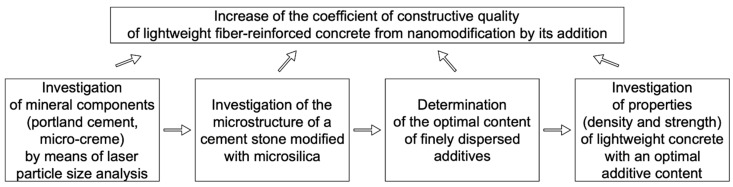
Structural and methodological scheme of experimental research.

Purpose of the work: increasing the coefficients of constructive quality for all types of strengths of lightweight fiber-reinforced concrete due to its modification with micro silica. Experimental studies were carried out following the structural and methodological scheme shown in Figure 1.

After formulating the hypothesis, scientific novelty, substantiating the relevance, identifying the goal, and setting goals, we will determine the necessary set of materials, equipment, and methodological apparatus for conducting the experimental and analytical part of the study.

## 2. Materials and Methods

### 2.1. Materials

During the research, additives-free Portland cement of the PC 500 D0 brand was used, the physical and mechanical characteristics and chemical composition of which are presented in Table 1.

Granite crushed stone was used as a sizeable dense aggregate, and slag pumice was used as a porous one. The physical and mechanical characteristics of a large dense and porous aggregate are presented in Table 2 and Table 3.

Quartz sand was used as a fine aggregate, the physical characteristics of which are presented in Table 4.

For sieves with a mesh size of 2.5, 1.25, 0.63, 0.315 and 0.16, the grain size composition of the sand is given, where the upper line is the partial sieve rest, and the lower line is the full sieve rest.

Glass fiber pretreated with surfactant was used as dispersed reinforcement. Table 5 shows the physical and mechanical characteristics of the fiber used.

Micro silica grade MS-85 was used as a reaction-chemical pozzolanic additive. Table 6 shows the chemical composition of micro silica MS-85.

Polycarboxylate superplasticizer MELFLUX 5581 F manufactured by BASF Construction Additives (Krasnodar, Russia) was used as a plasticizing additive in an amount of 0.3% by weight of cement.

As a control composition, heavy concrete of class B30 was designed with the workability of the mixture corresponding to the draft of the cone 1–4 cm [30]. The content of coarse aggregate fractions is represented by the following ratio: 60–fraction 10–20 mm; 40–fraction 5–10 mm [31]. The parameters of the composition of the concrete mixture obtained as a result of calculations are reflected in Table 7.

During the manufacture of lightweight fiber-reinforced concrete, part of the volume of dense aggregate was replaced with the same volume of porous in an amount of 40%. Glass fiber was introduced in the amount of 3% by weight of the cement. The water consumption was adjusted until the required concrete mix mobility was obtained [32].

### 2.2. Methods

When dispersed mineral additives are introduced, their uniform distribution over the volume is very important, especially in the case of the manufacture of heavy and lightweight concretes [33,34].

The mixture of powders was processed in a homogenizer to increase the homogeneity of the binder (cement and additives).

**Figure 2:**
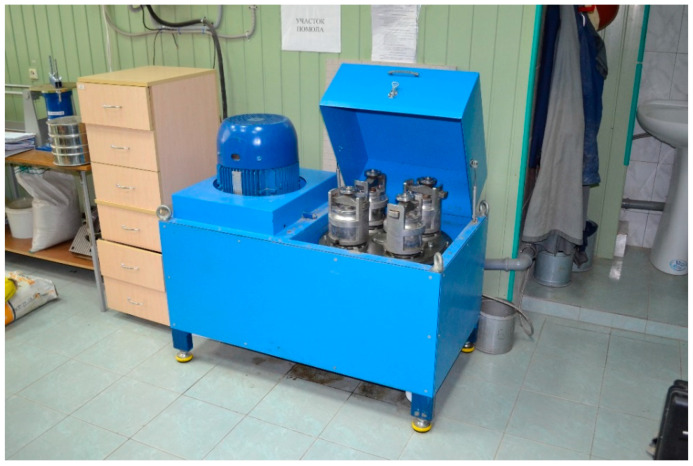
Planetary mill “Activator-4M”.

Homogenization of Portland cement and micro silica was carried out in a planetary ball mill “Activator-4M”. General view of the planetary ball mill “Activator-4M” is shown in Figure 2, and its technical characteristics are presented in Table 8.

For the directed organization of the microstructure of the cement stone, the ratio of the diameters of the mineral additive (filler) and the binder *d_F_/d_B_* < l is considered favorable when the filler particles serve as a substrate onto which the products of neoplasms diffuse. In this case, diffusion is possible both through the dispersion medium and through the surface of the solvation shells. Furthermore, the outflow of the substance into the contact zone prevents the early overlap of the surface of the binder grains by nuclei, which should cause a deepening of the hydration processes.

Determination of the granulometric characteristics of powdery raw materials (Portland cement, MS) was carried out using the method of laser granulometry. This method allows to determine particles size and their percentage in the material, as well as to study their shape and morphology.

**Figure 3:**
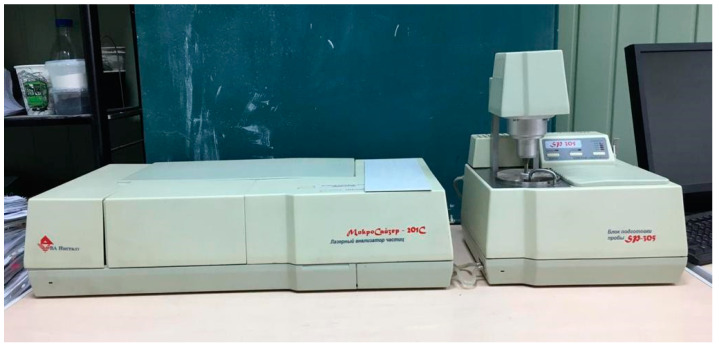
Laser particle analyzer Microsizer 201C.

Microsizer 201C laser particle analyzer was used to carry out the particle size analysis of the mineral components. It is a fully automated device designed for fast and accurate measurement of particle size distribution in the range of 0.2–600 µm. The general view of the device is shown in Figure 3, and its technical characteristics are shown in Table 9.

The study of the microstructure was carried out on a VEGA II LMU scanning electron microscope (Tescan, Brno, Czech Republic) at an accelerating voltage of 20 kV.

Images were obtained using SE detectors. The SE (Secondary Electron) detector provides information on the surface morphology of the sample. The BSE detector (reflected or backscattered electrons) provides information about the phase and chemical inhomogeneity of the material (phases and areas with a higher average atomic weight are colored in lighter shades). The surface of the samples was sputtered with metal in an Emitech sputtering device.

The experimental research program is presented in Table 10.

Also used in this research:

− technological equipment: laboratory concrete mixer BL-10 (LLC “ZZBO”, Russia, Chelyabinsk region, Zlatoust); laboratory vibrating platform SMZh-539-220A (LLC “IMASH”, Armavir, Russia);
− testing equipment: hydraulic press IP-1000 (LLC NPK TEKHMASH, Neftekamsk, Republic of Bashkortostan, Russia); tensile testing machine R-50 (LLC “IMASH”, Armavir, Russia);
− measuring instruments: measuring metal ruler 500 mm; laboratory scales; device for measuring deviations from the plane NPL-1; device for measuring deviations from perpendicularity NPR-1.

In the course of the study, standard test methods for raw materials and products based on them were used.

Compressive and tensile flexural strength tests were carried out by GOST 10180 “Concretes. Methods for strength determination using reference specimens” [35].

When testing for compression, sample cubes are installed with one of the selected faces on the lower base plate of the testing machine (press) centrally relative to its longitudinal axis, using the marks drawn on the plate of the testing machine.

After placing the sample on the support plates of the testing machine, align the top plate of the testing machine with the upper support face of the model so that their planes adjoin entirely one another. The sample is loaded to failure at a constant rate of load rise (0.6 ± 0.2) MPa/s.

**Figure 4:**
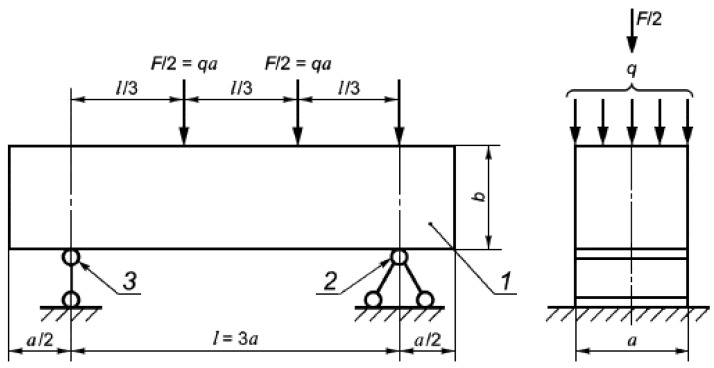
Flexural tensile test setup (a—width and height of the sample; F—load; q—distributed load; l—span; 1—sample; 2—articulated fixed support; 3—articulated-movable support).

The prism specimen is installed in the testing machine according to Figure 4 and loaded to failure at a constant rate of load growth (0.05 ± 0.01) MPa/s.

When testing for axial tension, the sample is fixed in a tensile machine and loaded to failure at a constant rate of load growth (0.05 ± 0.01) MPa/s.

The prismatic strength was determined following the requirements of GOST 24452 “Concretes. Methods of prismatic, compressive strength, modulus of elasticity and Poisson’s ratio determination” [36].

When determining the prismatic strength of concrete, loading the specimen to a load level equal to (40 ± 5)% should be performed in steps equal to 10% of the expected breaking load, keeping the loading rate (0.6 ± 0.2) MPa/s.

At each stage, load should be held for 4 to 5 min (during heating-up to 15 min) and the readings on the instruments at the beginning and at the end of the load stage should be recorded in the test log.

At a load level equal to (40 ± 5)%, remove the instruments from the sample, unless there are other requirements stipulated by the test program. After removing the devices, further loading of the sample should be carried out continuously at a constant speed in accordance with the requirements of GOST 10180-2012.

The values of the coefficients of constructive quality (*CSQ*) for different types of strengths were calculated using the following formulas:

(1) $$CSQ_{Rb,cub}=\frac{R_{b,cub}}{\rho }$$

where $R_{b,cub}$ is cubic compressive strength, MPa; *ρ* is the density of concrete, g/cm^3^.

(2) $$CSQ_{Rb}=\frac{R_{b}}{\rho }$$

where $R_{b}$ is the prismatic compressive strength, MPa.

(3) $$CSQ_{Rbtb}=\frac{R_{btb}}{\rho }$$

where $R_{btb}$ is tensile strength in bending, MPa.

(4) $$CSQ_{Rbt}=\frac{R_{bt}}{\rho }$$

where $R_{bt}$ is the axial tensile strength, MPa.

Having formed the experimental-methodological apparatus and setting the parameters of the study, we present the results obtained by us in the course of our work.

## 3. Results

**Figure 5:**
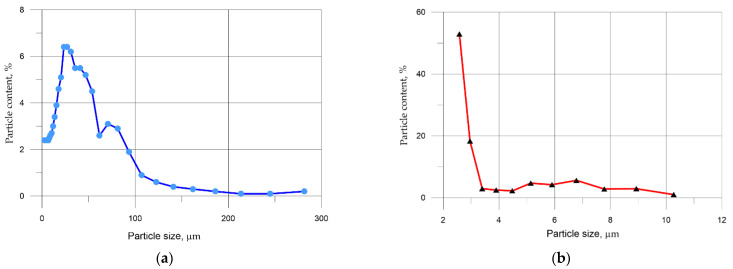
Particle size distribution of (**a**) Portland cement, (**b**) micro silica particles.

The granulometric analysis of the mineral components used—Portland cement and micro silica—is presented in Figure 5.

Based on micro silica granulometric composition shown in Figure 5, it follows that the main share (52.9%) falls on particles with a size of up to 2.58 inclusive and 47.1% for particles with the size of up to 10.27 microns. For cement (Figure 4), the maximum mass fraction (49.4%) falls on particles with a length from 17.8 to 53.7 μm, and particles with a size of up to 10 μm—24.3%. In this case, the average particle diameter of Portland cement is 31 microns, and micro silica—3.6 microns, respectively.

The results obtained from the tests of prototype cement beams with different percentages of micro silica are presented in Table 11.

From Table 10 it can be seen that the maximum values of the strength characteristics were recorded for samples of cement stone with a 10% content of silica fume additive.

As known, active mineral finely dispersed additives are capable in the presence of water interacting with portlandite—$Ca{\left(OH\right)}_{2}$ at ordinary temperatures, forming compounds with astringent properties—CSH, according to the reaction scheme:

(5) $$SiO_{2}\;\left(amorphous\;in\;mineral\;additives\right)+Ca{\left(OH\right)}_{2}+H_{2}O\to CSH$$

So, during the hydration of cement in the composition of hydrated neoplasms of cement stone, CSH and free CH are of the most significant importance of the occupied volume and effect on the properties of the cement stone. The formation of these compounds is represented by the following scheme of chemical reactions occurring during cement hydration:

(6) $$C_{3}S,\;C_{2}S,\;C_{3}A\;and\;C_{4}AF+water\to CSH,\;CAH\;and\;CFH+CH$$

where $C_{3}S$ is alite $\left(3CaOxSiO_{2}\right)$;

$C_{2}S$ is belite $\left(2CaOxSiO_{2}\right)$;

$C_{3}A$ is tricalcium aluminate $\left(3CaOxAl_{2}O_{3}\right)$;

$C_{4}AF$ is tetra calcium alumoferrite $\left(4CaOxAl_{2}O_{3}xFe_{2}O_{3}\right)$;

CH is calcium hydroxide;

CSH is calcium hydrosilicates;

CAH is calcium hydroaluminates;

CFH is Calcium Hydroferrites.

**Figure 6:**
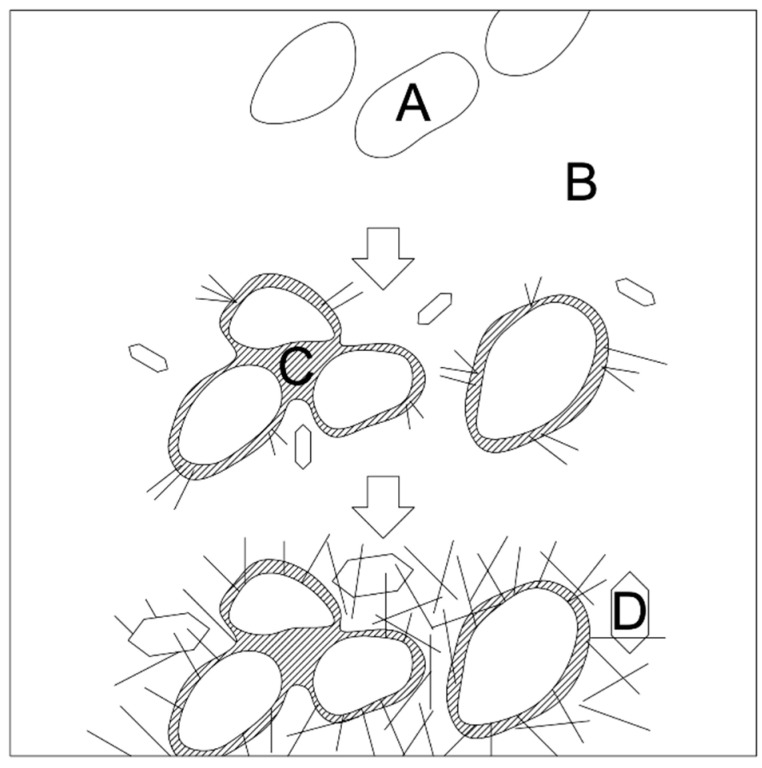
Hydration of Portland cement in the absence of micro silica addition (A—Portland cement; B—water; C—CSH; D—CH).

Figure 6 shows a diagram of the hydration of Portland cement, which does not contain active mineral additives.

Calcium hydrosilicates are compounds that provide concrete strength. Calcium hydroxide (15–30% of the total volume of hydration products) is a substance that has a loose structure and can dissolve in water, therefore it does not play an important role in increasing the strength and durability of concrete.

**Figure 7:**
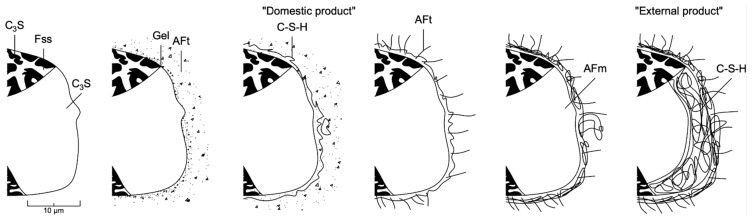
Development of the microstructure of cement stone in the process of hydration of Portland cement, where: Fss—calcium aluminoferrite; AFt—calcium hydrosulfoaluminate; AFm—calcium hydrosulfoaluminoferrite.

Clinker hydration products with a low degree of crystallinity increase the strength of the cement stone. Therefore, to increase the density of cement stone, it is necessary to create such a structure that contains many fine-crystalline “internal products” CSH by reducing the proportion of larger-crystalline “external products” CSH (see Figure 7).

The addition of silica fume binds free CH into less soluble and denser compounds, which makes it possible to reduce the thickness and increase the density of the transition zone in concrete. Therefore, due to this pozzolanic reaction, the content of free $Ca{\left(OH\right)}_{2}$ in the form of large crystals of portlandite decreases, while the content of CSH increases, increasing the strength and durability of concrete by increasing the density and strength of both the cement stone itself and the contact zone between cement stone and aggregate grains in the structure of high-strength concrete.

**Figure 8:**
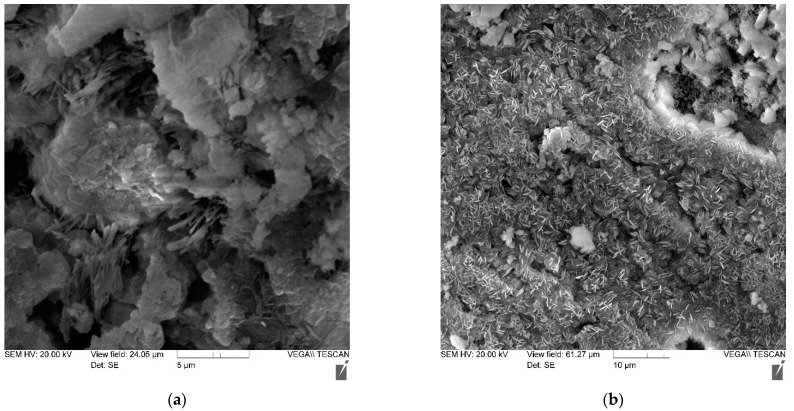
Photo of the microstructure of a cement stone without additives: (**a**) with 2000× magnification (**b**) with 1000× magnification.

**Figure 9:**
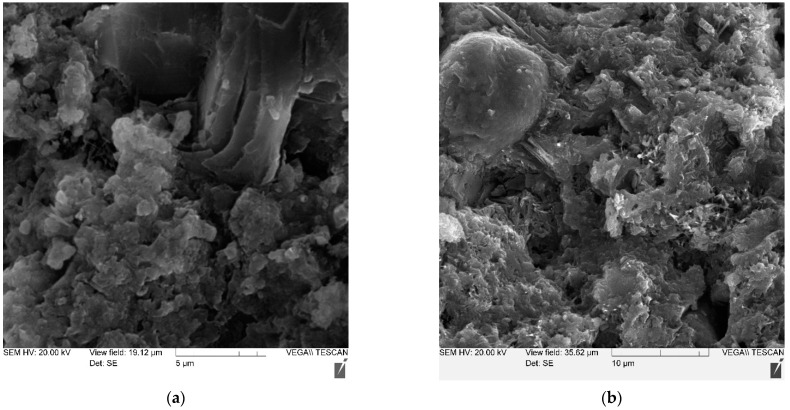
Photo of the microstructure of a cement stone with an MS-85 additive in an amount of 6%: (**a**) with 2000× magnification (**b**) with 1000× magnification.

**Figure 10:**
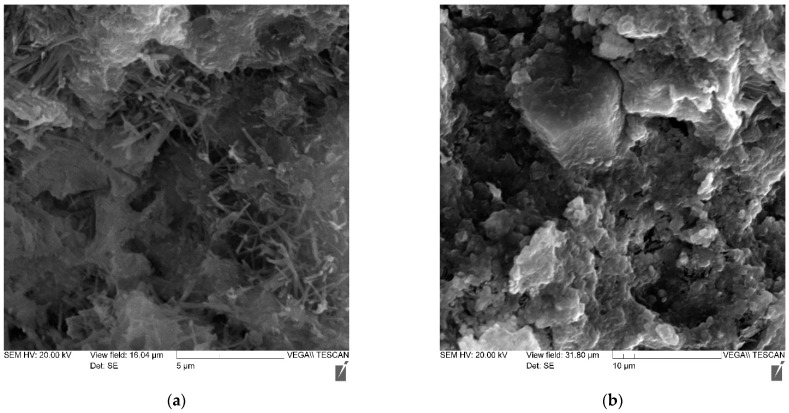
Photo of the microstructure of a cement stone with an MS-85 additive in an amount of 10% and an additive of MELFLUX 5581 superplasticizer: (**a**) with 2000× magnification (**b**) with 1000× magnification.

**Figure 11:**
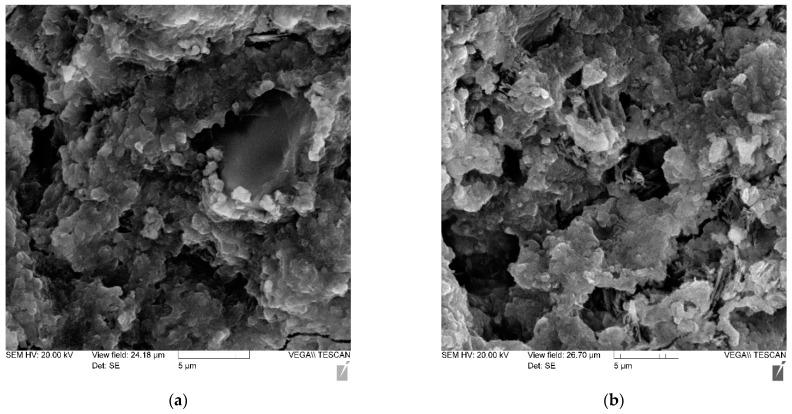
Photo of the microstructure of a cement stone containing an MS-85 additive in an amount of 12% and an additive of MELFLUX 5581 superplasticizer: (**a**) with 2000× magnification (**b**) with 1000× magnification.

Using the method of electron microscopy, a comparative analysis of the effect of micro silica and a complex of micro silica (MS) + superplasticizer (SP) on the formation of the structure and properties of cement stone was carried out. Pictures of the main compositions are shown in Figure 8, Figure 9, Figure 10 and Figure 11—(a) with 2000× magnification, and (b) with 1000× magnification.

Thus, the study of cement stone chips in an electron microscope made it possible to draw the following conclusions:

- the structure of non-additive cement stone is heterogeneous, has a block character and is represented by weakly crystallized interlayers of highly basic calcium hydrosilicates, including portlandite accumulations;
- the addition of MS contributes to the formation of a denser homogeneous structure, preferably from low-basic calcium hydrosilicates;
- the joint introduction of MS and SP is accompanied by the formation of a dense structure, represented by both a weakly crystallized and a gel-like phase, in which portlandite is practically not detected.

**Figure 12:**
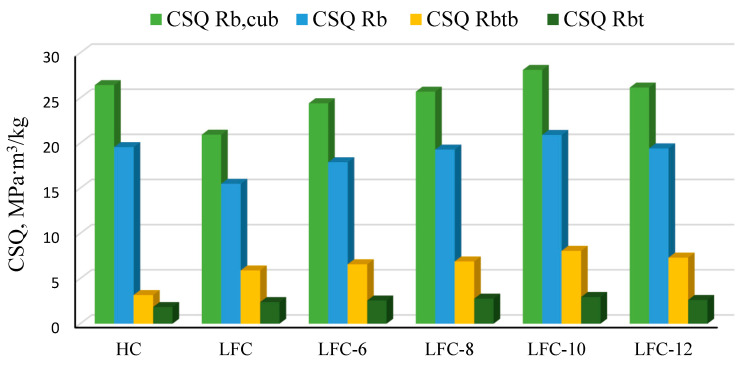
Values of structural quality factors for various types of concrete (HC—heavy concrete; LFC—lightweight fiber concrete; LFC-6—lightweight fiber concrete with MS = 6%; LFC-8—with MS = 8%; LFC-10—with MS = 10%; LFC-12—with MS = 12%).

The results obtained from the tests of prototypes of lightweight fiber-reinforced concrete with different percentages of micro silica are presented in Table 12 and Figure 12.

After analyzing the obtained experimental data on the effect of the percentage of micro silica additive on the strength characteristics of lightweight fiber-reinforced concrete, the following was established:

- the maximum germination of cubic strength is observed in the prototypes of lightweight fiber-reinforced concrete with a micro silica content of 10% (MS = 10%); so in comparison with samples of lightweight fiber-reinforced concrete without additives, it was 35%; a similar comparison was made for other compositions: so the increase for samples with MS = 6% was 17%, for samples with MS = 8–23%, for samples with MS = 12–25%;
- the increase in prismatic compressive strength for specimens with MS = 6% was 16%, for specimens with MS = 8–25%, for specimens with MS = 10–35%, and for specimens with MS = 12–26%;
- the increase in tensile strength in bending for specimens with MS = 6% was 12%, for specimens with MS = 8–17%, for specimens with MS = 10–37%, and for specimens with MS = 12–24 %;
- the increase in axial tensile strength for specimens with MS = 6% was 8%, for specimens with MS = 8–16%, for specimens with MS = 10–24%, and for specimens with MS = 12–10%.

Figure 12 shows that the maximum values of the structural quality factors, calculated for various types of strength, are observed in lightweight fiber-reinforced concrete with MS = 10%.

So, in comparison with heavy concrete, the increase was:

▪ $CSQ_{Rb,cub}$: 6%;
▪ $CSQ_{Rb}$: 7%;
▪ $CSQ_{Rbtb}$: 253%;
▪ $CSQ_{Rbt}$: 60%.

In comparison with lightweight concrete without MK-85 additive, the increase was:

▪ -$CSQ_{Rb,cub}$: 34%;
▪ -$CSQ_{Rb}$: 35%;
▪ -$CSQ_{Rbtb}$: 37%;
▪ -$CSQ_{Rbt}$: 24%.

The coefficient of constructive quality (*CSQ*) for tensile strength in bending for lightweight fiber-reinforced concrete with MS = 10% is two and a half times higher than that for heavy concrete without additives due to the complex influence of the following factors:

(1) the addition of fiber, which significantly increases the tensile strength in bending.
(2) replacement of a part of the dense aggregate with a porous one, which leads to a significant reduction in the mass of concrete products with a slight loss of strength;
(3) replacing part of the cement with lighter micro silica.

After carrying out experimental studies, processing the results obtained and performing the necessary calculations, as well as after completing the theoretical interpretation with the help of high-precision microscopic equipment of the physical picture of the processes of structure formation, a thorough analysis and comparison of the results obtained with the results of other authors should be carried out.

## 4. Discussion

The analysis of the results obtained showed the high efficiency of the proposed recipe, technological and design solutions. From the formulation point of view, the possibility of nanomodification of lightweight fiber-reinforced concrete with micro silica was established, as a result of which, firstly, the expediency of such nanomodification, and secondly, the dosage of the content of nano-modifying additives of micro silica in such concrete was determined to obtain the highest characteristics.

From a technological point of view, the compatibility of low-density dispersion-reinforced concrete with a nano-modifying additive of micro silica was tested. The revealed possibility of simple technological mixing of such components and the test results proved that the joint work of fiber-reinforcing fiber and hardening micro silica is beneficial to the resulting concrete and gives a synergistic effect.

From a constructive point of view, the possibility of dispersed reinforcement of such lightweight fiber-reinforced concrete, which, in turn, at the level of a cement stone, is nano-modified with the addition of micro silica, was determined.

This is how you can describe the qualitative picture of the study.

From a quantitative point of view, it was revealed that the optimal value of the percentage of nano-modifying additives of micro silica will be a value equal to 10%.

At the same time, the mechanism of interaction between the nano-modifying additive and the structure of the forming concrete has been studied. A high packing density of particles in such concrete was proved, and the addition of micro silica contributed to better hydration of the cement stone, its more perfect structure, and a decrease in pore space. Such an additive influenced the pore structure at the micro level, while an important factor was the simultaneous dispersed reinforcement of concrete, which ultimately turned out to be light in weight and at the same time possessing significant strength compared to analogs. In order to identify operational prospects, it was also necessary to evaluate the effectiveness of the proposed recipe, technological and design solutions not in terms of absolute indicators, but in terms of relative ones, in terms of the coefficient of constructive quality. Their effectiveness was proved due to the fact that the coefficient of constructive quality increased due to a significant increase in strength, while the mass of structures practically did not change from the introduction of a nano-modifying additive.

To assess the effectiveness of the developed proposals, it is important to conduct a comparative analysis with the results previously obtained by other authors. As already noted in the Introduction section, various authors [9,10,11,12,13,14,15,16,17,18,19,20,21,22,23,24,25,26,27,28,29] considered the issues of nanomodification of heavy concrete with various additives. We, for the first time, carried out a study of the possibility of using a nano-modifying additive, namely micro silica for dispersed reinforced concretes, among other things, having a light weight. For the first time, we studied the effect of a combination of factors of nanomodification of the structure of a cement stone, which implies the work of the additive at the micro level, and at the macro level—dispersed reinforcement with fibers. Thus, by hardening concrete at the micro and macro levels, we get a result that is different from the results of the authors who previously worked on this issue.

The novelty of the research is the application of such a combined modification of concrete at the micro and macro levels, and thus the research is original, different from the previously performed works.

For the first time, we obtained new fundamental knowledge about the formation of the microstructure of fiber-reinforced concrete with a lightweight of nanomodified micro silica additive and developed ideas about the joint work of nanomodified cement stone with dispersed fiber in lightweight fiber-reinforced concrete with an increased coefficient of structural quality.

## 5. Conclusions

According to the results of the literature review and analysis of sources devoted to research carried out on the topics of nano-modified concretes, it was revealed that studies aimed at increasing the coefficients of the constructive quality of lightweight fiber-reinforced concrete due to its modification with micro silica were not carried out earlier.

The effect of nano-modifying additives of micro silica on the strength characteristics of lightweight fiber-reinforced concrete was theoretically and experimentally established. Thus, the introduction of micro silica additive into Portland cement, having an average grain size of 3.6 μm, in an amount of 10% of the cement mass in combination with a superplasticizer, is the most effective. This effect of the additive is due to the fact that it acts, as it were, as a substrate for the crystallization of hydrated neoplasms.

Large-scale experimental studies were carried out, compositions were selected, new samples of materials obtained for the first time were molded.

It is proposed to evaluate the effectiveness of each accepted recipe-technological solution in terms of relative indicators—the calculated values of the structural quality coefficients for all experimentally determined strength characteristics of lightweight fiber-reinforced concrete modified with the addition of micro silica. The coefficient of constructive quality for tensile strength in bending of lightweight fiber-reinforced concrete with MK = 10% is two and a half times higher than that of heavy concrete without additives and up to 37% higher than that of lightweight fiber-reinforced concrete without additives.

The results obtained are recommended for practical application in construction and design, and are also the basis for further scientific fundamental and applied research.

## Figures and Tables

**Table 1:** Physical and mechanical characteristics of Portland cement PC 500 D0 and its chemical composition.

Indicator Title	Value
**Physics and Mechanics**	
Compressive strength at the age of 28 days, MPa	54.8
Setting time, min -start -the end	155 220
Fineness of grinding, passage through a sieve No. 008, %	96.7
Specific surface, m^2^/kg	331
Normal density of cement paste, %	23.5
**Chemical**	
Weight loss on ignition, %	0.70
Silicon oxide content (SiO_2_), %	20.89
Aluminum oxide content (Al_2_O_3_), %	4.72
Iron oxide content, (Fe_2_O_3_), %	4.32
Calcium oxide content (CaO), %	63.27
Magnesium oxide (MgO), wt %	2.45
Sulfuric acid anhydride (SO_3_), wt %	2.81
Alkaline oxides in terms of Na_2_O, wt %	0.69
Free calcium oxide content (CaO_fr_), %	0.00
Chloride ion (Cl^−^), wt %	0.038
Insoluble residue, %	0.20

**Table 2:** Physical and mechanical characteristics of crushed granite.

Fraction	Bulk Density, kg/m^3^	True Density, kg/m^3^	Crushing, wt %	Content of Lamellar and Needle-Shaped Grains, wt %	Voidness, %
5–20	1437	2620	11.4	8.1	45

**Table 3:** Physical and mechanical characteristics of slag pumice.

Fraction	Bulk Density, kg/m^3^	True Density, kg/m^3^	Strength by GOST 32496-2013, MPa	Voidness, %
5–20	612	1310	0.8	53

**Table 4:** Physical characteristics of dense fine aggregate.

Grain Composition							Passing through a Sieve No 0.16, wt. %	Fineness Modulus	Content of Dust and Clay Particles, %	True Density, g/cm^3^	Bulk Density, kg/m^3^
Sieve Size, mm											
Partial and Full Sieve Rest, %											
10	5	2.5	1.25	0.63	0.315	0.16					
0	0	0.17	1.39	8.86	45.80	41.03	2.49	1.66	1.1	2650	1438
		0.17	1.56	10.42	56.21	97.25	99.74				

**Table 5:** Physical and mechanical properties of fiber.

Glass Fiber	Tensile Strength, MPa	Fiber Diameter, μm	Fiber Length, mm	Elastic Modulus, GPa	Density, kg/m^3^	Elongation to Break,%
	3100	13	12	72	2600	4.6

**Table 6:** Chemical composition of micro silica MS-85.

Material	Oxide Content, %							
	SiO_2_	Al_2_O_3_	Fe_2_O_3_	CaO	MgO	R_2_O	SO_3_	Loss on Ignition
МК-85	82.3	1.7	3.0	1.1	0.2	0.8	3.5	7.4

**Table 7:** Parameters of the composition of the concrete mixture.

Indicator Title	W/C	PC, kg/m^3^	W, l/m^3^	CS, kg/m^3^	S, kg/m^3^	ρ_cm_, kg/m^3^
Indicator value	0.58	327	190	1315	573	2405

Note: W/PC—water-cement ratio, W—water consumption, CS—crushed stone consumption, S—sand consumption, ρ_cm_—concrete mix density.

**Table 8:** Technical characteristics of “Activator-4M”.

Indicator Title	Units	Value
Planetary disc: -rotational speed -effective diameter	rpm mm	100–800 400
Rotation speed of drums	rpm	150–1650
Centrifugal acceleration	m/s^2^	1500
Drums	pieces	4
Drum volume	mL	1000
Loading balls	g	600–1400
Powder loading	g	50–400
Steel grade (balls)		ShH15SG
Drums sizes	mm	Ø95 × 180

**Table 9:** Technical characteristics of Microsizer 201C.

Indicator Title	Units	Value
Particle size range	μm	0.2–600
Radiation source		He-Ne laser
Detector		Photodiode Array
Number of registration channels	pieces	38
Sample preparation system		Ultrasonic Disperser
Sample chamber volume	ml	50
Ultrasonic frequency	kHz	50
Ultrasonic power	W	Up to 200

**Table 10:** Experimental research program.

Research Type	Samples Number
Investigation of the microstructure of a cement stone modified with micro silica	Cement beam without additives, size 40 × 40 × 160 mm–3 pcs.
	Cement beam with MS-85 additive in the amount of 6%, size 40 × 40 × 160 mm–3 pcs.
	Cement beam with MS-85 additive in 10% and addition of MELFLUX 5581 superplasticizer, size 40 × 40 × 160 mm–3 pcs.
Investigation of the properties (density and strength) of lightweight concrete with MS-85 additives	A total of 6 series of samples were made: heavy concrete; lightweight fiber-reinforced concrete without adding MS-85; lightweight fiber-reinforced concrete with MS-85 additive in the amount of 6%; lightweight fiber-reinforced concrete with MS-85 additive in the amount of 8%; lightweight fiber-reinforced concrete with MS-85 additive in the amount of 10%; lightweight fiber-reinforced concrete with MS-85 additive in the amount of 12%. Each series of samples contains: cubes 100 × 100 × 100–3 pcs; prisms with dimensions 100 × 100 × 400 mm–9 pcs.

**Table 11:** Results of tests for strength in compression and tensile in bending of cement bar specimens with different MS content.

Composition	Compressive Strength, MPa	Tensile Strength in Bending, MPa
C	55.3	6.4
C + 6% MS-85	59.8	7.1
C + 10% MS-85 + SP	64.7	7.8
C + 12% MS-85 + SP	61.3	7.4

**Table 12:** Test results of prototypes of heavy concrete and lightweight fiber-reinforced concrete with different percentages of micro silica.

Concrete Characteristics	Heavy Concrete	Lightweight Fiber Concrete				
		МS-85 Content, wt % by Cement Mass				
		0	6	8	10	12
Density, kg/m^3^	2414	2087	2097	2089	2095	2091
Cubic compressive strength, MPa	63.8	43.7	51.2	53.7	58.9	54.7
Prismatic compressive strength, MPa	47.2	32.3	37.5	40.3	43.8	40.6
Flexural tensile strength, MPa	7.7	12.3	13.8	14.4	16.9	15.3
Axial tensile strength, MPa	4.5	5.0	5.4	5.8	6.2	5.5

## Data Availability

The study did not report any data.
